# Spatial statistical modelling of capillary non-perfusion in the retina

**DOI:** 10.1038/s41598-017-16620-x

**Published:** 2017-12-01

**Authors:** Ian J. C. MacCormick, Yalin Zheng, Silvester Czanner, Yitian Zhao, Peter J. Diggle, Simon P. Harding, Gabriela Czanner

**Affiliations:** 1 Department of Eye & Vision Science, Institute of Ageing and Chronic Disease, University of Liverpool, 6 West Derby Street, Liverpool, L7 8TX United Kingdom; 2 Malawi-Liverpool Wellcome Trust Clinical Research Programme, Queen Elizabeth Central Hospital, Blantyre, Malawi; 3 Centre for Clinical Brain Sciences, University of Edinburgh, Edinburgh, United Kingdom; 4 School of Computing, Mathematics and Digital Technology, Faculty of Science and Engineering, Manchester Metropolitan University, Manchester, M1 5GD United Kingdom; 5 Cixi Institute of Biomedical Engineering, Ningbo Institute of Industrial Technology, Chinese Academy of Sciences, Ningbo, China; 6 CHICAS, Lancaster Medical School, Lancaster University, Lancaster, LA1 4YB United Kingdom; 7 St Paul’s Eye Unit, Royal Liverpool University Hospital, Liverpool, L7 8XP United Kingdom; 8 Department of Biostatistics, Institute of Translational Medicine, University of Liverpool, 1-5 Brownlow Street, Liverpool, L69 3GL United Kingdom

## Abstract

Manual grading of lesions in retinal images is relevant to clinical management and clinical trials, but it is time-consuming and expensive. Furthermore, it collects only limited information - such as lesion size or frequency. The spatial distribution of lesions is ignored, even though it may contribute to the overall clinical assessment of disease severity, and correspond to microvascular and physiological topography. Capillary non-perfusion (CNP) lesions are central to the pathogenesis of major causes of vision loss. Here we propose a novel method to analyse CNP using spatial statistical modelling. This quantifies the percentage of CNP-pixels in each of 48 sectors and then characterises the spatial distribution with goniometric functions. We applied our spatial approach to a set of images from patients with malarial retinopathy, and found it compares favourably with the raw percentage of CNP-pixels and also with manual grading. Furthermore, we were able to quantify a biological characteristic of macular CNP in malaria that had previously only been described subjectively: clustering at the temporal raphe. Microvascular location is likely to be biologically relevant to many diseases, and so our spatial approach may be applicable to a diverse range of pathological features in the retina and other organs.

## Introduction

**Figure 1:**
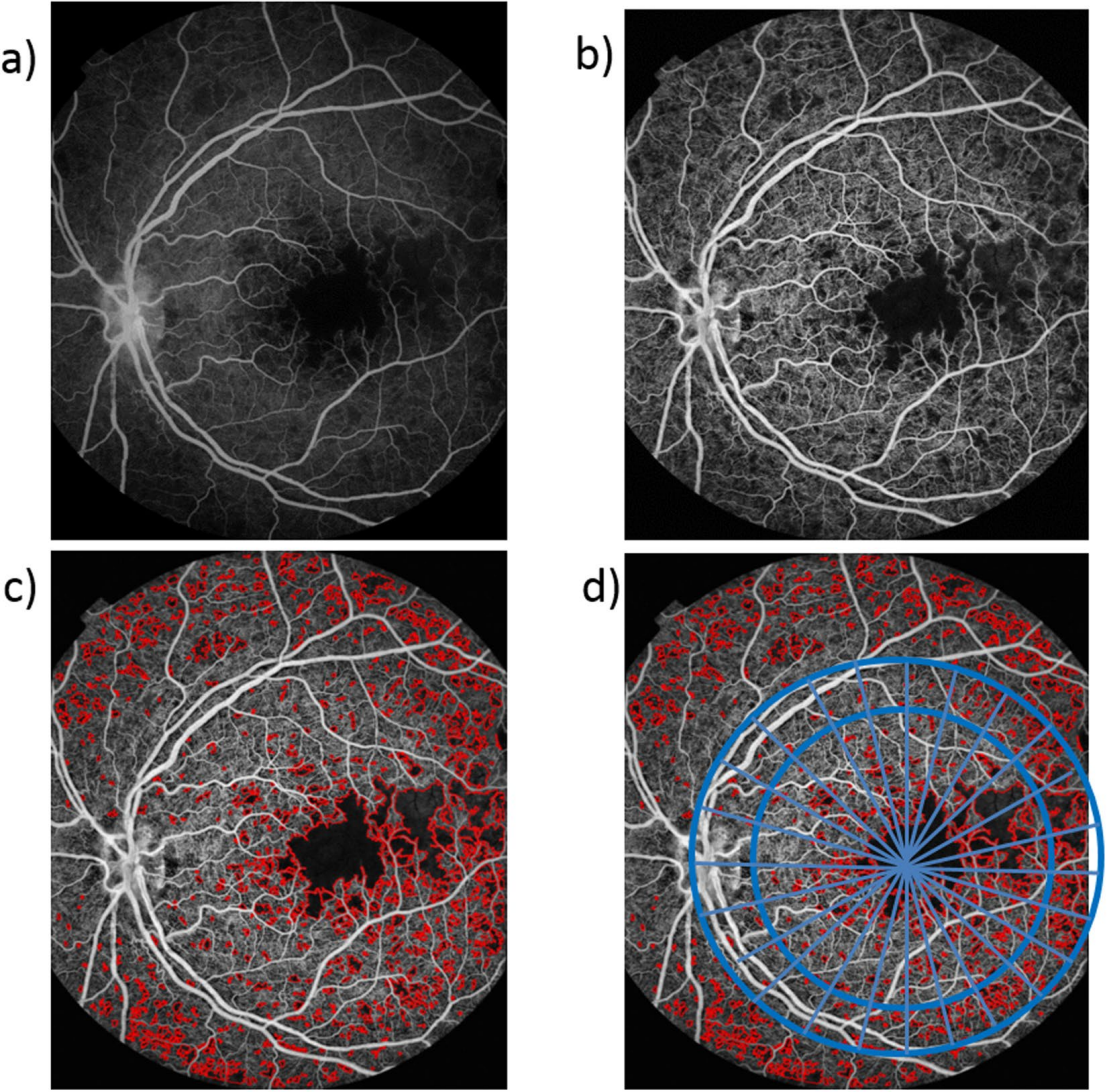
Analysis of an FA image. (**a**) The original FA image, (**b**) Enhanced image with improved contrast, (**c**) Texture-based segmentation was applied to detect CNP, (**d**) A grid imposed over the image allowed the proportion of CNP in each area to be calculated.

The retinal microcirculation is exquisitely accessible to clinical observation, and unlike other organs, the retinal vasculature is arranged perpendicular to an optical axis. Consequently alterations to small vessel flow can be easily mapped using techniques such as fluorescein angiography (FA) and optical coherence tomography angiography (OCT-A). Capillary non- perfusion (CNP) appears as distinctive dark areas with geographic boundaries, and develops when blood fails to reach areas of the capillary bed (Fig. 1a–c). It is a feature of several major causes of blindness including diabetic maculopathy, retinal vein occlusion, and retinal artery occlusion^1^. CNP also occurs in malarial retinopathy, and can be graded manually according to a validated scheme^2^. Malarial retinopathy is seen in children and adults with cerebral malaria, which has a high mortality rate. The retina and brain sustain similar damage in cerebral malaria, and several retinal signs are associated with death (reviewed in^3^).

As with grading schemes for retinal vein occlusion^4,5^ the malarial retinopathy grading scheme assesses the overall area of CNP in various large retinal regions. Manual grading is necessarily semi-quantitative, time consuming and costly. These and other constraints mean that manual grading is impractical for large image datasets, and at best captures only a tiny fraction of the biological information contained in a retinal image.

Beyond lesion type, frequency, and area, spatial characteristics may be a particularly relevant aspect of a retinal image. This is because the retinal microvasculature is not homogenous but rather composed of regions with different vascular topology, geometry, and corresponding haemorheology and physiology. For example, the foveal avascular zone is a unique region where the retina is supplied solely from the underlying choriocapillaris. The perifoveal region has one capillary layer, which forms an oxygen diffusion watershed with the choriocapillaris. The temporal macula and horizontal raphe contain a further watershed between superior and inferior temporal arcades^3^. Therefore, the biological meaning of CNP in one sub-region of the macula may be different from that of a lesion of similar size in an adjacent region. Current grading techniques are too coarse to allow such distinctions.

There are several automated methods for segmenting areas of CNP from retinal images^6,7^, including one developed by our group and applied to a subset of manually-graded images from children with malarial retinopathy^2,8^. Automated segmentation provides the user with CNP metrics, such as the proportion of CNP-pixels in a given area, and has advantages over manual grading in terms of cost and reproducibility. However, as with the Likert scales of manual grading schemes, the raw metrics produced by automated segmentation may not necessarily describe lesions in the most biologically relevant way. Therefore, we need new analytical tools to refine image segmentation data into biologically meaningful information. Such information would have uses as an outcome measure in clinical trials for diabetic maculopathy and retinal vein occlusions, as well as clinical practice.

A spatial statistical model could be a good candidate for this data-information interface. After all, a retinal image is not unlike an areal photograph of geographical features such as roads, buildings, or rivers – and spatial models have been successfully applied to geographical problems^9^. However, the standard concept of geographical spatial modelling cannot be directly applied to analyses of medical images, because biological tissue has an implicit biological structure that must be respected. In the case of retinal angiography this structure can be regarded as two-dimensional. Retinal OCT provides three-dimensional information, as does MRI of other organs such as the brain^10^. An ideal spatial model must account for the biological topography of the tissue, and must be flexible to be adapted to specific problems of the image acquisition.

One approach is to analyse the distribution of pixels within the whole image. However, pixels do not necessarily form the basis for clinical interpretation, and this may miss the underlying anatomical and physiological context^11^. Instead, a sector-wise approach can be used based on clinically meaningful sub-regions. For example, Bowman and Waller^11^ developed model for the heart based around a physiological model of the left ventricle containing 20 distinct sectors. Similarly George *et al*.^12^ proposed a model of 16 sectors for the left ventricle. Lange^13^ proposed a linear model with patterned correlated errors. Bowman and Kilts^14^ proposed correlation maps to display distribution of correlation across the brain, but this does not allow for varying qualities of images, and for missing parts of an image. To the best of our knowledge there are very few spatial methods for analysing the output of retinal segmentation algorithms in terms of microvascular location, such as by Gadde *et al*.^15^.

In this paper, we present a new spatial model of CNP based on a linear mixed effect framework. It enables robust, accurate, and fast characterisation of segmentation data. We have applied this analytical approach to a cohort of images of malarial retinopathy since these have abundant macular CNP and have both corresponding manual grading data and automated segmentation.

## Methods

### Overview of our framework

A standard way to study CNP from a retinal angiogram is to divide it into gradable regions. A 50 degree retinal angiogram image of the posterior pole usually captures the macula as well as a consistent area of surrounding retina. CNP can therefore be graded within two regions: an inner circle (the macula), and an outer ring that includes the disc, temporal vascular arcades, and temporal raphe. A human grader typically evaluates the extent of CNP by giving a score on ordinal scale from 0 to 4.

To refine this approach, we: (i) extend this approach into a spatially refined grid on the retinal images; (ii) automatically create a spatially resolved CNP profile for each image; (iii) find the best statistical model that characterises the spatially resolved profiles and their association with clinical outcome. Then to demonstrate the utility of our spatially resolved approach we compare it with simple overall CNP index.

### Image dataset

To illustrate our spatial framework, we analysed CNP in retinal images from study of cerebral malaria. The study includes patients within an ongoing survey of severe malaria in the Paediatric Research Ward (PRW) of Queen Elizabeth Central Hospital (QECH), Blantyre, Malawi^16^. Informed consent was given by the parents or guardians of all patients. This research adhered to the tenets of the Declaration of Helsinki, and was approved by the ethics committee at the University of Malawi College of Medicine and at collaborating academic institutions (Michigan State University or the Liverpool School of Tropical Medicine).

A sequence of 50-degree images were taken after pupil dilation with Tropicamide 1% and Phenylephrine 2.5%, using a Topcon 50-EX optical unit (Topcon, Tokyo, Japan) and Nikon E1-H digital camera. The image size is 3008 × 1960 pixels. For each patient we have intentionally chosen a single image of the study eye (left eye unless data non-available) to create this dataset. We included all patients with malaria from 2006 to 2010 who had a 50 degree FA image obtained at the time of admission (Supplementary Figure 1), except for cases where images were of poor quality. Manual grading involved dual grading with adjudication, by professional graders masked to clinical information and the other grader’s scores. A third expert adjudicated score discrepancies. Graders evaluated the quality of each image (poor, fair, good, excellent) according to the visibility of capillaries around the foveal avascular zone (Supplementary Figure 2). They graded the extent of macular CNP on ordinal scale (0, 1, 2, 3, 4) according to pre-defined standard images. Further details of image acquisition and manual grading are in ref.^2^. The unit of analysis for manual grading is the retina. For automated analysis we selected the best quality image of the macula for all eligible subjects, and we consider only one retinal image per patient.

### Image pre-processing and segmentation

This was done in a series of steps. First, the centres of the optic disc and the fovea were manually localised by professional graders by following a protocol for manual grading of retinal features in cerebral malaria^8^. The distance between these structures was assumed to be 2.5 optic disc diameters (ODD), and the optic disc (OD) was assumed to have a diameter of 1,800 um. Then, CNP regions were automatically segmented by a selective segmentation method^8^ (Fig. 1c). In brief, the field of view of the image under consideration was detected by using the Otsu thresholding technique and morphological operators, the image was then enhanced by a top hat filter for better contract. A new texture based variational segmentation model was applied to the enhanced image to produce CNP candidate regions. The final CNP segmentation was achieved by applying a pre-trained Adaboost classifier to the candidate CNP regions. This automatic segmentation technique has been evaluated against expert ophthalmologists’ manual annotations on both malarial retinopathy and diabetic retinopathy datasets with good performance (8). After the automatic segmentation step, each pixel Each FA image of about 10^6^ pixels in an FA image will be deemed as either CNP pixel or not.

### Creating spatially resolved CNP profiles

**Figure 2:**
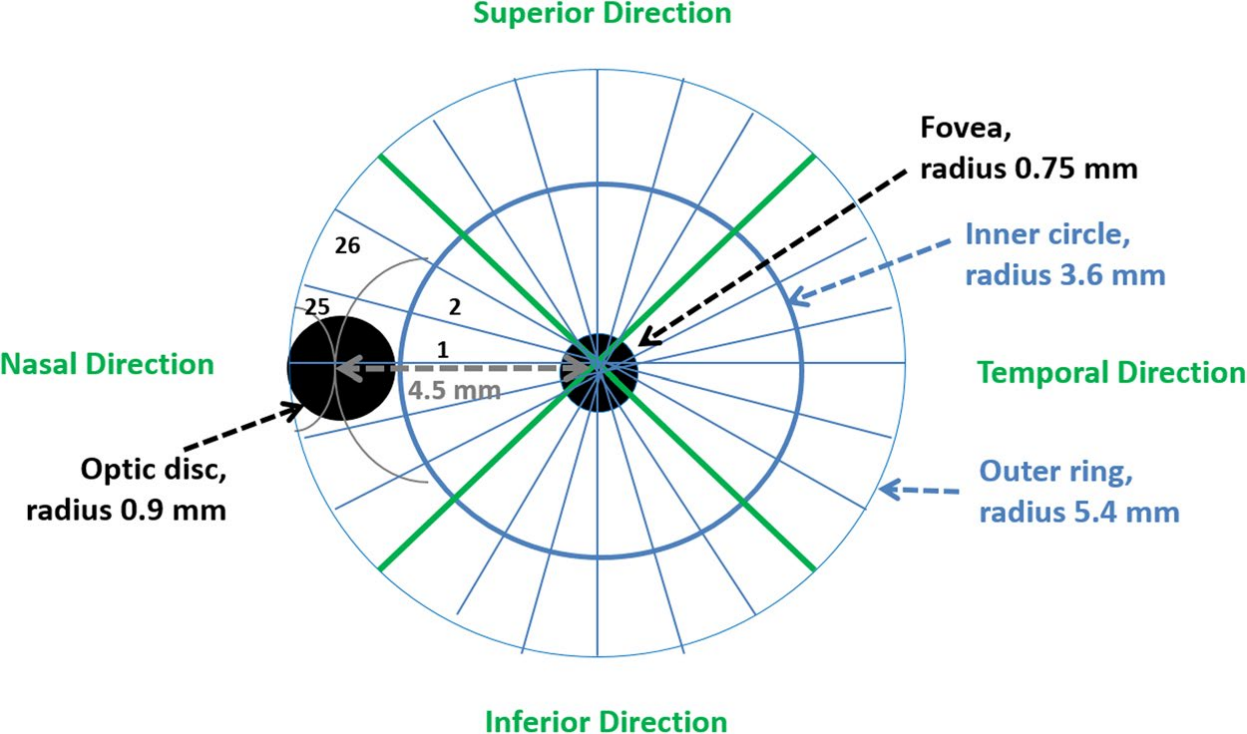
The division of the macula into segments, illustrated on a drawing of the left eye. The macula is divided into two circular areas (an inner circle and an outer ring), and each of these is further divided into 24, thus producing 48 sectors. The inner circle contains sectors 1 to 24, and the outer ring contains sectors 25 to 48. Sectors are numbered upwards from the disc, starting with the inner circle. Thus sectors 1,2,3 and 22,23 24 represent the nasal quadrant; sectors 4–9 and 28–33 represent the superior quadrant; sectors 10–15 and 34–39 temporal quadrant, and sectors 16–21 and 40–45 the inferior quadrant. The orientation for a right eye is just the mirror image - with sectors 1 and 25 on the right of the panel, instead of the left of the panel, so that they still overlap the optic disc.

For each segmented image we created a spatially resolved CNP profile. To characterise each CNP segmented image, we superimposed a grid of 48 sectors (Figs 2, 1c) onto each segmented image of retina (Fig. 1d). In order to obtain consistent segment arrangement for the left and right eye, the segment was indexed in clockwise order for the left eye while in anti-clockwise order for the right eye. The inner circle corresponds to the anatomical region of macula, defined as a circle centred on the foveola with a radius equal to the distance between the foveola and the temporal edge of the optic disc. The macula has radius 20*0.1*OD = 2*1,800 = 3.6 mm. The outer ring corresponds to radius of 20*0.1*1,800 um = 3.6 mm to 30*0.1*1,800 um = 5.4 mm, and extends approximately 1.8 mm beyond the inner circle. Then for each CNP segmented image we calculated the proportion of CNP-pixels for each of 48 sectors, leading to a vector of 48 values on a continuous scale from 0 to 100. This is the automated spatially resolved CNP damage profile.

For comparison we also calculated automated overall CNP index as the simple proportion of CNP-pixels separately for the inner circle and outer ring (continuous scale 0 to 100%). We compared the manual CNP grade of the inner circle, which is just one number measured on an ordinal scale (1, 2 or 3), with the automated overall CNP index of the inner circle, which is one number (on scale of 0 to 100%) via means (two-sample t-test), medians (Mann-Whitney U test) and consistency (Intraclass Correlation Coefficient, ICC).

### The spatial statistical model for spatially resolved CNP profiles

The spatially resolved CNP profiles are 48-dimensional vectors. Therefore to compare them effectively across the two outcome groups we built a mixed effect model with spatial correlations. The model describes the spatial distribution of CNP across the retina by specifying the mean CNP in each of 48 sectors. The inclusion of spatial correlation in the calculation for each sector results in a smoother spatial mean CNP profile, which in turn leads to smaller standard errors and increased power of statistical comparisons than would be obtained by treating the mean CNP values as 48 free parameters. To assure the continuity between sectors we chose sine and cosine harmonic functions as basis functions because they are naturally defined on a circular system. We begin here by introducing the general model for the *i* th image and then use it to formulate the model for all images collectively.

The model is based on following assumptions

- One eye per patient, and one time point only, one image per eye (i.e. no replications), indexed by $i=1,\ldots ,I;$
- Images have varying qualities: excellent, good, or fair, and indexed by $q=1,2;$
- Some parts of the images may be missing;
- Data from retina may have a complex correlation structure that must be taken into account in the model;
- Each image has 24 directions (Fig. 1d). The directions in image are indexed by $d=1,\ldots ,24$;
- Each image has two rings. The rings are indexed by $r=1,2$, where $r=1$ is for inner circle and $r=2$ is for outer ring;
- The outcome group values are indexed by $g=1,2$; where $g=1$is for survival with full recovery or with sequelae and $g=2$ is for death.

Then the general form of the linear mixed effect model assumes that the variability seen in the individual sectors is due to three effects: variation common to all retinas, variation specific to the retina (hence subject) and random variation. If we denote the CNP for retina *i*, direction *d*, ring *r* as ${Y}_{idr}$ then ${{\boldsymbol{Y}}}_{i}$ (48 × 1) represents the vector of CNP pathological damage profile for image (retina or subject) *i*. Then the linear mixed effect model can be written as

1 $${{\boldsymbol{Y}}}_{i}=f({{\boldsymbol{X}}}_{i},{\boldsymbol{\beta }})+{{\boldsymbol{Z}}}_{i}{{\boldsymbol{w}}}_{i}+{{\boldsymbol{e}}}_{i}$$

where ${\boldsymbol{\beta }}\,(2{\rm{k}}\times 1)$ is the fixed effects parameter vector, ${{\boldsymbol{X}}}_{i}(48\times 2{\rm{k}})$ is the design matrix for fixed effects, f(.) are basis functions (sine and cosine), ${{\boldsymbol{Z}}}_{i}(48\times 2q)$ is the design matrix for random effects. The fixed effects, $f({{\boldsymbol{X}}}_{i},{\boldsymbol{\beta }})$, in Eq. 1 describe the values of CNP on the level of all subjects (i.e. the between subject differences), the random effects, ${{\boldsymbol{Z}}}_{i}{{\boldsymbol{w}}}_{i}$, describe the values of CNP at the level of individual subjects (i.e. the within-subject differences).

We modelled the spatial correlation between measurements from different sectors for a given image. In general spatial covariance model the magnitude of correlation is inversely related to the distance between sectors. We calculated distances in the 48-sectors (Figs 1d or 2) by assuming that the 24 sectors of the inner circle have radii equal to 1.8 mm (=3.6/2) and the 24 sectors of the outer ring have radii equal to 2.75 mm (=5.4/2). To calculate distances between any two sectors, we used reference locations of each sector with polar coordinates $(m,\alpha )$, where

- for sectors 1–24, $m=1.8$ and $\alpha =2\pi /24,4\pi /24,$ $6\,\pi /24,\,$…, $\,24\,\pi /24,$
- for sectors 1–24, $m=2.75$ and $\alpha =2\pi /24,\,4\pi /24,$ $6\,\pi /24,\,$…, $\,24\,\pi /24.$

Next, we performed a model selection procedure to find the specific form of the linear mixed effect model that gives the best fit to the imaging CNP data. We applied a stepwise method to fit the model: the first step was to choose the fixed effects first on the basis of the Akaike Information Criterion (AIC), the second step was to add the random effects via analysis of variance, and the third step was to select the spatial correlation structure. The fit was finally confirmed using standard goodness-of-fit residual analysis. The final chosen covariance model was a Gaussian spatial correlation model as the suitable correlation structure for the spatial sectors, related inversely to the distance between sectors. The best selected model is:

2 $$\begin{array}{rcl}{Y}_{idr} & = & \sum _{g=1}^{2}{\beta }_{g}{I}_{g}+\sum _{g=1}^{2}\sum _{r=1}^{2}\sum _{j=1}^{5}{\beta }_{grj}^{sin}sin(2\pi jd/24){I}_{grd}\\  &  & +\,\sum _{r=1}^{2}\sum _{j=1}^{5}{\beta }_{grj}^{cos}cos(2\pi jd/24){I}_{grd}\\  &  & +\,\sum _{g=1}^{2}{w}_{gi}{I}_{g}+{e}_{idr},\end{array}$$

3 $$[\begin{array}{c}{w}_{i}\\ {e}_{i}\end{array}]\sim N([\begin{array}{c}0\\ 0\end{array}],[\begin{array}{cc}{\sigma }_{w}^{2}{I}_{2\times 2} & 0\\ 0 & {\sigma }_{q}^{2}{V}_{e}\end{array}])$$

where ${\beta }_{g}\,$is the intercept for group *g*, ${I}_{g}$ is an indicator function for group *g*, ${I}_{grd}$ is an indicator function for group *g*, ring *r* and direction *d*. In total 10 goniometric functions were considered but the first five had the best AIC so others were dropped. The values ${w}_{gi}$ in Eq. 2 represents random intercepts for two disease outcome groups and it has a diagonal variance covariance matrix. The vector of random errors ${e}_{i}\,(48\times 1)$ follows a multivariate normal distribution with autocorrelation structure with common correlation coefficient and with the variance modelled as depending on the quality of the image: we allow different variability for excellent, good and fair quality images; and these variabilities are free parameters estimated with the model by maximising the likelihood.

The model was fitted using the restricted maximisation likelihood in the R statistical package (function lme). There are several challenges when fitting this model to the CNP segmented images. Firstly, some parts of images were missing due to the nature of the FA. We used the best macular FA images taken on the patients. Such images may show only the beginning of the periphery, and the image may not be perfectly centred. Both characteristics can lead to missing CNP values at the image boundaries. Conveniently, the mixed effect model has the advantage of utilizing all CNP data from all available areas of retina. Such an analysis assumes that the parts of image are missing at random and using all available data from all subjects.

### Data availability statement

The data that support the findings of this study are available from the MRet study investigators but restrictions apply to the availability of these data, which were used under license for the current study, and so are not publicly available. Data are however available from the authors upon reasonable request and with permission of co-author SPH.

## Results

### Patients

Between 2006 and 2010, 161 patients with malarial retinopathy had admission FA. Of these, we excluded 29 from the analysis because image quality was graded as poor. Our dataset therefore includes images from 132 eyes in 132 patients (Supplementary Figure 1).

### The automated overall CNP index correlates with manually graded CNP

Manual grading is an imperfect reference standard, yet it is important to directly compare our automatic quantification of lesions with manual evaluation. We made two comparisons. Firstly, in all 132 images, the inner circle (i.e. macula) automated overall CNP was found to be positively associated with manual grading of macular CNP (Supplementary Figure 3a, p = 0.11 two-sample t-test, p = 0.05 Mann-Whitney U test, n = 87 and 45) and gave excellent consistency (ICC = 0.88, p < 0.01). Secondly, in excellent quality images, the inner circle automatic CNP score was positively correlated with the inner circle manual CNP (Supplementary Figure 3b, p-value = 0.02 two-sample t-test and 0.03 Mann-Whitney U test, n = 24 and 21) and gave excellent consistency (ICC = 0.89, p < 0.01).

### The automated overall CNP index increased with death in our cohort, but not significantly

In images with fair to excellent quality the automatically calculated inner circle CNP is 19.8% and 20.2% in the survived and death group, respectively, but this difference is not significant (p = 0.65, two-sample t-test, Table 1). The outer-ring simple automatic CNP is 8.8% and 8.9% in the survived and death group, respectively, which is again not significant (p = 0.88, two-sample t-test, Table 1). In excellent images, the inner circle simple automatic CNP is 18.9% and 19.3% in the survived and death group (p = 0.72, two-sample t-test, Supplementary Table 2). For the outer ring, in excellent images, the CNP is 7.2% and 8.2% in the survived and death group respectively (p = 0.27, two-sample t-test, Supplementary Table 2).

**Table 1:** Associations of simple overall CNP measures vs death using images of excellent, good and fair quality (n = 132).

**Overall CNP index in images of excellent, good or fair quality (n = 132)**		**Automatic grading:** Total percentage of CNP damaged pixels: One number for inner circle and one number for outer ring	
		Continuous scale, %	p-value
Inner circle	Survived	Mean = 19.8 SD = 4.3	p = 0.73 (Logistic regression) p = 0.65 (2-sample t-test)
	Died	Mean = 20.2 SD = 2.8	
Outer ring	Survived	Mean = 8.8 SD = 3.5	p = 0.86 (Logistic regression) p = 0.88 (2-sample t-test)
	Died	Mean = 8.9 SD = 3.3	

### Spatially resolved CNP profiles show directional distribution of CNP in images

**Figure 3:**
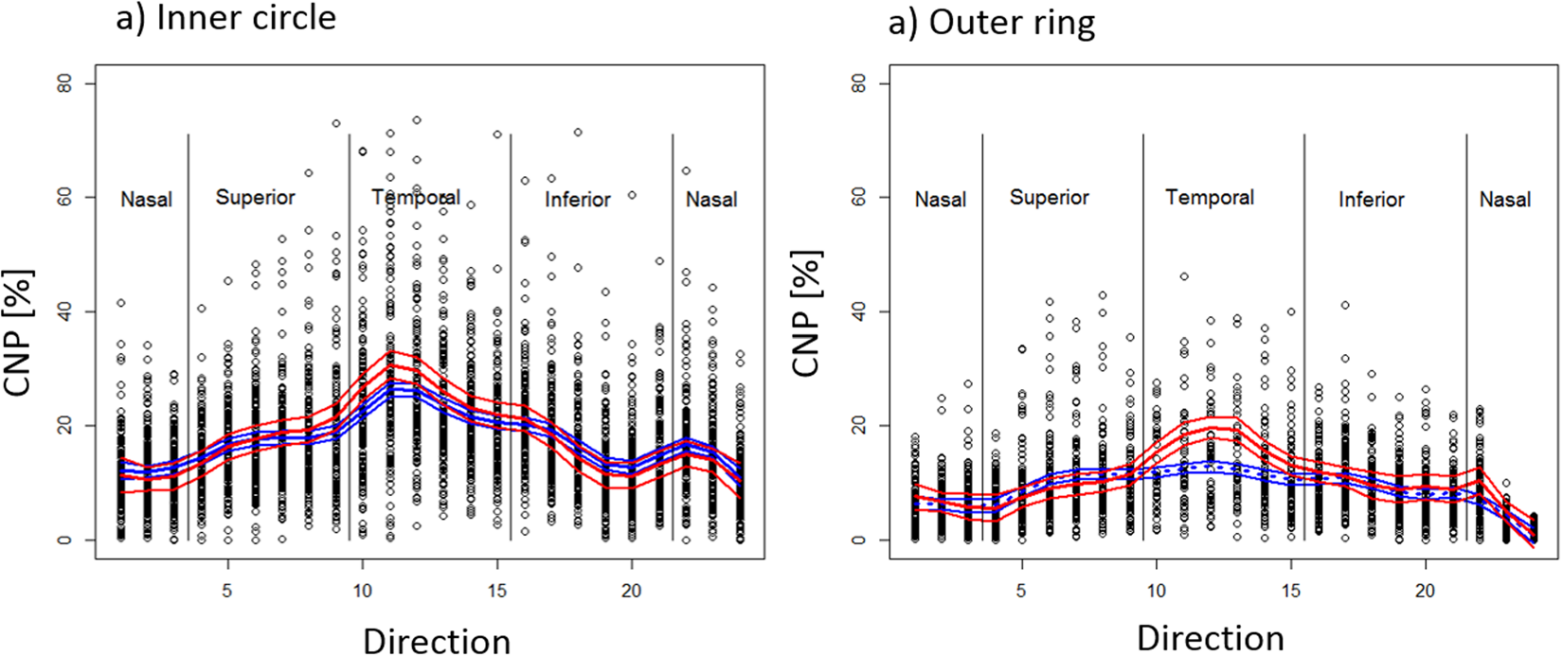
Values of CNP in 132 images in the inner circle and outer ring. Mean values were calculated using LOESS fits (span = 0.25, Cleveland, 1979) and 95% confidence intervals were calculated using bootstrap for those who survived (blue) and died (red).

We found that CNP was more severe in the temporal macula compared to other segments (Table 2, Direction effect, p < 0.001). This trend is supported by raw means (without spatial smoothing, Fig. 3). Such can be also seen in multiple t-test comparisons (Fig. 3c,d, green colour). This is consistent with agreement between spatial CNP and previously unquantifiable subjective clinical observations about CNP^3^.

**Table 2:** Sources of CNP variations across disease groups and space in mixed effect model with spatial correlation.

**Associations using all images and using spatially detailed model of retinal CNP damage**		Num df	Den df	F-statistic	P-value
**Source of variation**					
Fixed effects	Intercept	1	5743	2588.0175	<0.001
	Direction	9	5743	124.7424	<0.001
	Overall group effect	1	152	36.66	0.026
	Group effect in inner circle	1	152	135.15	<0.001
	Group effect in outer ring	1	152	132.44	<0.001
	Ring	1	5743	1064.2291	<0.001
	Direction*Group	9	5734	1.9585	0.030
	Direction*Ring	9	5743	10.0754	<0.001
	Group*Ring	1	5743	2.6297	0.105
	Direction*Group*Ring	9	5743	1.0979	0.360
Random effect	Retina (Between subject variation), SD	2.849			
Random term	Within subject variation, SD	6.621			
Spatial correlation	Gaussian, Range	0.520			
Image quality	1 Excellent	1.000			
	2 Good	1.212 (multiple of SD)			
	3 Fair	1.350			

The effect of direction was modelled via 5 sine and cosine functions as a fixed effect. The ring and group are dichotomous (ring is either inner cirle or outer ring), hence there were 10 parameters estimated for each combination of the levels of the factors.

**Figure 4:**
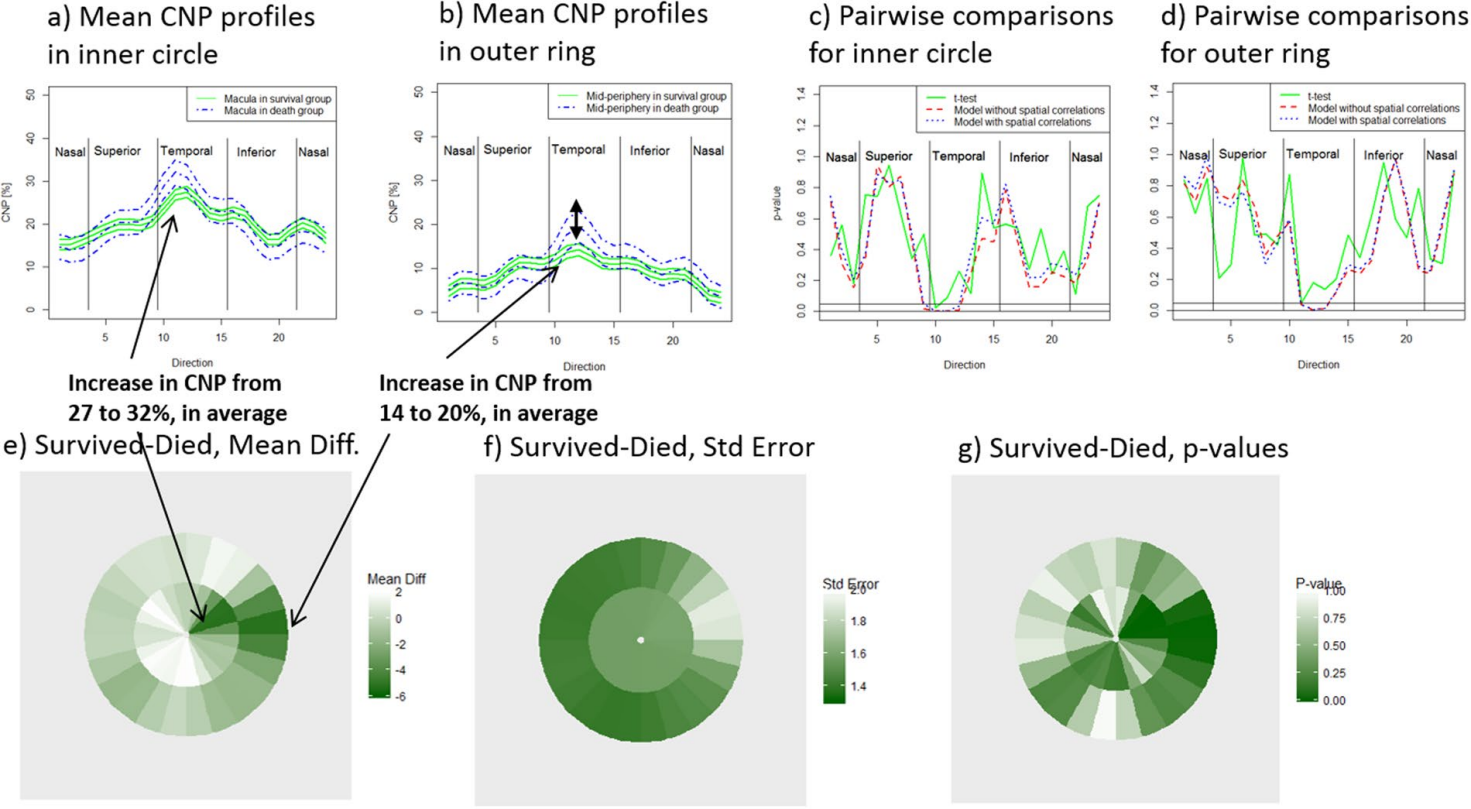
Comparison of CNP between subjects who survived and died using the spatial mixed-effect model. The children who died show (**a**) higher mean CNP in temporal direction in inner circle, (**b**) higher CNP in temporal direction in outer ring. The p-values of pairwise comparisons are more decisive in the spatial model compared to t-test (**c** and **d**). The mean CNP difference of survived-died as calculated from the spatial model (**e**) in a circular plot are higher in temporal direction. The standard errors of mean differences calculated from the spatial model (**f**) are smaller than for t-test. The p-values calculated from the spatial model are significant in temporal direction (**g**).

We further investigated which directional segments were most important by using the mixed effect model to estimate the mean directional CNP profiles with 95%CI (Fig. 4). We first examined variation arising from spatial location and clinical outcome (Table 2), and found that the amount of CNP depends on the distance from the fovea and also on the direction from the fovea (Table 2, main effect of direction and ring, both p < 0.001). Then we found that the directional profiles have a similar shape, but the temporal segments are elevated in subjects who died (Fig. 4a,b). In both the inner and outer rings the temporal segments have the highest differences in CNP between outcome groups (respectively: 27% to 32%, p = 0.014; 14% to 20%, p = 0.003 Table 2; see also Fig. 4c,d).

Overall, our spatial analysis points to the horizontal raphe (corresponding to the temporal segments of the inner and outer rings) as an especially important biological site for severe CNP.

### The spatially resolved CNP profiles are associated with death

Using the mixed effect model with spatial correlations, we found that CNP differs between subjects who live and die (overall group effect: p = 0.02, Table 2). This is in contrast with the simple overall automatic inner-ring CNP was slightly increased in death in our cohort but not significantly. This is not surprising since there is a large amount of noise in sectors with very subtle signal in the temporal direction that gets lost when averaging the CNP across all directions (Fig. 3). Spatially resolved CNP provides a stronger basis for inference compared to a simple proportion of CNP over the whole region.

### The variability of the spatially resolved CNP profile increases with lower image quality

Spatially resolved CNP does appear to also contain important information about image quality. We found that some profiles vary more (over their own mean CNP) than others. We investigated if this variability correlates with the image quality which was manually assessed by human graders. We found that the variability of the CNP values increases with decreasing quality of the image in the inner circle (Supplementary Figure 4a, p-value < 0.001 ANOVA, n = 132) and also in the outer ring (Supplementary Figure 4b, p = 0.012 ANOVA, n = 132). This is consistent with increasing estimated variability of CNP between sectors. Within the mixed effect model we estimated the standard deviation of CNP in good and fair quality images to be 1.212 and 1.350 multiple of the SD in excellent quality image (Table 2). This suggests that the variability of a damage profile can be potentially used to develop an automated quality measure CNP images.

## Discussion

Evaluating macular CNP is an important step in clinical practice and research into new treatments for serious retinal diseases. Manual grading has several limitations: it is costly in terms of money and time, it is subjective, and it extracts only limited information from retinal images.

In this paper we propose a paradigm that brings two innovations: it replaces manual scores with automatic scores, and it interprets these within a spatially resolved profile of 48 sub-regions. This incorporates some of the biological structure of the macula into image analysis. We illustrate this spatial approach in the analysis of macular CNP in a study of 132 children with malarial retinopathy, and we show that: (i) automatically calculated CNP scores correlate with manually graded CNP (p = 0.03), (ii) in malarial retinopathy CNP generally occurs around the temporal macula (inner circle) and temporal raphe (outer ring) (p < 0.001) with 5% difference between survived and died groups (p < 0.001), (iii) using spatial information improves the power of our analysis when compared to non-topographical automatic grading and manual grading. Therefore, our framework offers an important improvement over manual grading. Our statistical spatial model can be computed using a mixed modelling framework (also called multilevel or hierarchical modelling), e.g.^17,18^, and public domain software for mixed-effect modelling that is readily available (e.g. R package nlme at https://cran.r-project.org/). We are preparing a dedicated software package that will be available on our webpage and plan to make it part of the R library.

Spatially resolved CNP gives improved statistical power to estimate associations with clinical outcome in paediatric CM. Furthermore, we were able to quantify a biological characteristic of macular CNP that had previously only been described subjectively: in malarial retinopathy macular CNP clusters around the temporal macula and temporal outer ring - an important watershed zone. This approach is likely to be useful for ongoing research into treatments for ischaemic retinal diseases, since it improves the accessibility and power of CNP as an outcome measure. It may also provide new information about relationships between the microanatomical location of CNP and various ischaemic disease processes in the retina, by providing spatial resolution that has previously been unavailable to investigators. For example, using our approach the spatial profiles of CNP in diabetic maculopathy and malaria could be compared, and interpreted according to vessel topology and sub-regional physiology.

The profile of 48 automated scores captures more information about lesions than the two simple automated scores or than traditional manual grading, as was seen in significant and stronger associations with outcome. As such, the profile of 48 scores better approximates what a clinician sees when looking at a retinal image, by including information about lesion size, frequency and also location. Manual grading for a given subject has the advantage that it includes information from a whole series of FA images, while automated techniques typically analyse only one image per patient. Our approach performs well despite this handicap. Extension to analyse a series of FA images is likely to improve performance further.

We illustrated our approach on data from malarial retinopathy, since this has a characteristic spatial distribution within the macula, and allowed us to compare our new approach to manual grading as a reference standard^2^ as well as an unambiguous outcome variable. Manual grading is often time consuming, is prone to inter-observer variation, and may not adequately capture important details such as the precise location of CNP lesions and their size. By overcoming some of the limitations of manual grading, automated detection of CNP can aid in more precise analyses of retinal disease and may potentially provide a more pragmatic option for assessment in clinical trials. While automated detection of CNP is well described^8^, to the best of our knowledge spatial interpretation of these data has not yet been attempted. We present a framework for automated interpretation of CNP, with the aim of quantifying an important retinal feature linked to fundamental disease processes in several retinal and systemic conditions.

There are limitations to our approach. These promising results largely rely on good CNP segmentation. This approach is not based on counting individual lesions. We decided to divide the macula into ‘pie-slice’ shaped sub-regions for pragmatic reasons, and because we were particularly interested in the temporal macula compared to the other macular sub-regions. Other sub-region shapes (for example, hexagons) may yield different results, and may be more suitable for studying other conditions. We evaluated our approach in a set of images from patients with malarial retinopathy. Future work could assess the pattern of CNP in other diseases, such as diabetic maculopathy. We compare our spatial approach to manual grading of retinal images. Manual grading is the result of a human viewing a whole series of images, while the spatial model is only applied to a single image. Furthermore, a human observer can assess image quality and select the most appropriate images during grading, while most automated techniques require image quality to be assessed prior to analysis. Future work could address these limitations by developing automated methods to select good quality macular images, and then apply the spatial model to the resulting series of images.

CNP is an important feature of several common retinal diseases and, importantly, a key outcome variable for randomised clinical trials. Our spatial technique is also relevant to other medical images, since the analytical limitations inherent in simply counting the type, number, and size of lesions apply to images in general, where such images contain a wealth of additional spatial information that is relevant to the disease in question.

We present a novel method for evaluating macular CNP based on the spatial distribution and extent of CNP. Spatial distribution is important because it is likely to contain information about regional variation in microvascular anatomy and physiology within the macula. Our analysis provides a way to interpret CNP segmentation data from automated algorithms while taking account of the topographical structure. Consequently, it can quantify topographical features of CNP noted subjectively by clinicians. Such a technique may also allow more powerful analyses of associations between macular CNP and clinical outcomes in basic research and clinical trials.

## Electronic supplementary material

Supplementary information
