# Supplementary material for: Spatial statistical modelling of capillary non-perfusion in the retina
Source: Sci Rep. 2017 Dec 1;7:16792. doi: 10.1038/s41598-017-16620-x (PMC5711887; doi:10.1038/s41598-017-16620-x)
Supplement: Supplementary file 1 — Supplementary information [file 41598_2017_16620_MOESM1_ESM.pdf]

Spatial statistical modelling of capillary non-perfusion in the retina

Ian MacCormick, Yalin Zheng, Silvester Czanner, Yitian Zhao, Peter J Diggle, Simon P Harding, Gabriela Czanner

Supplementary information

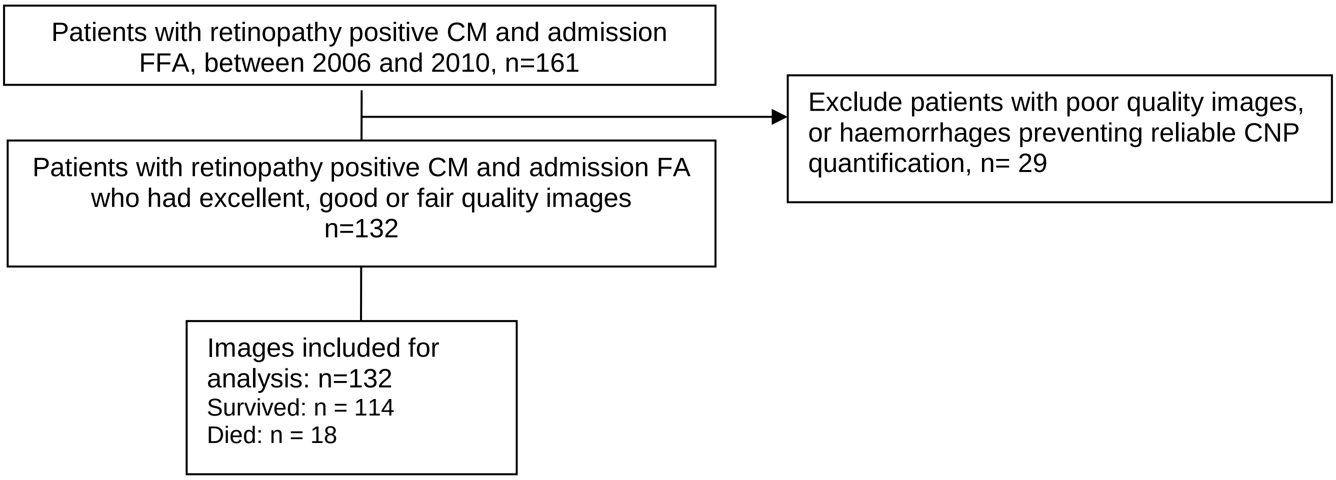

Figure 1. Derivation of the cohort of patients.

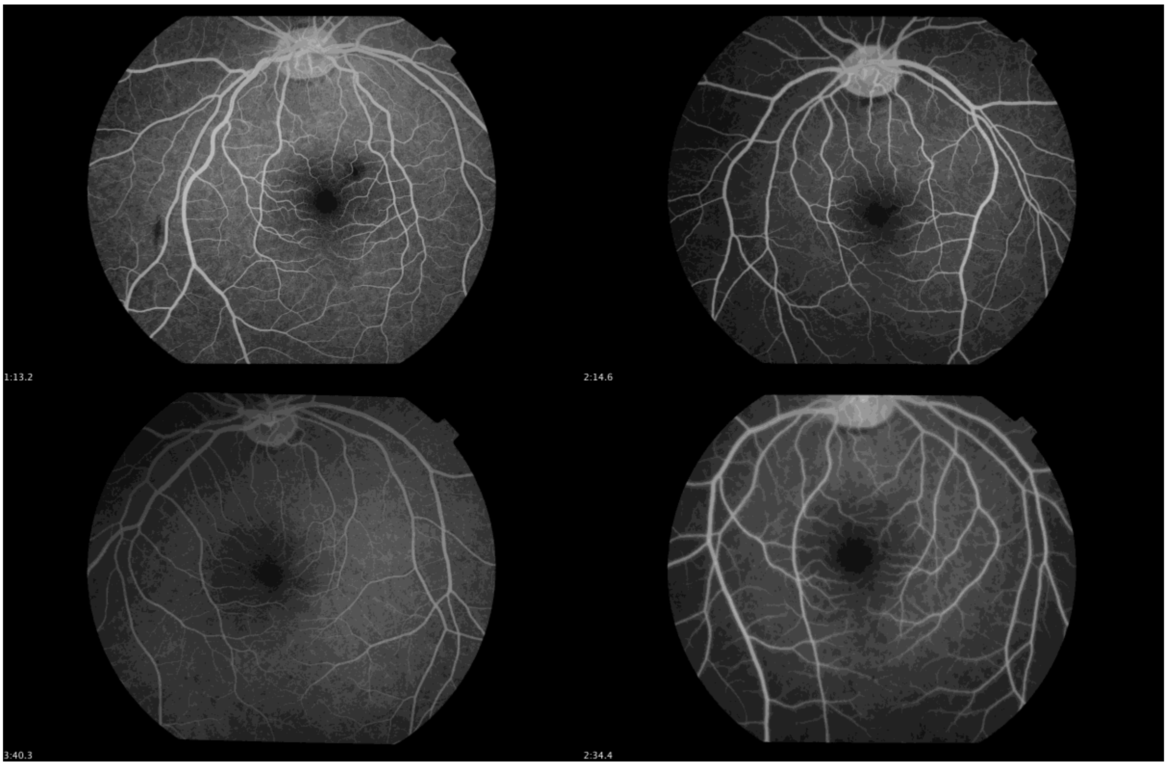

Figure 2. Images quality: Excellent (top left), good (top right), fair (bottom left) and poor (bottom right).

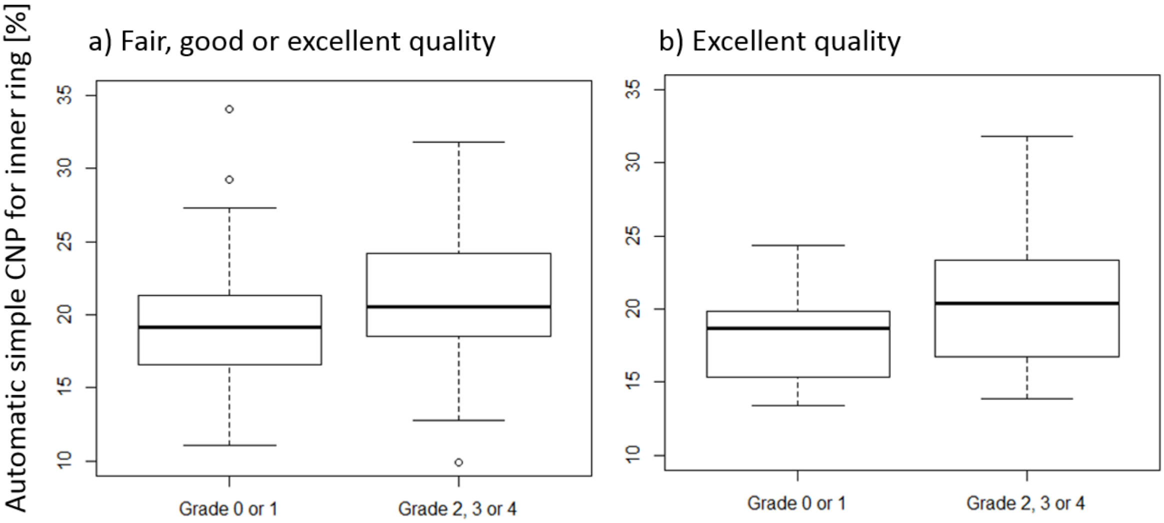

Figure 3 Comparison of automatic overall CNP in inner circle for images of fair, good or excellent quality (two-sample t-test p=0.11, Mann-Whitney U test p=0.05, n=87 and 45) and excellent quality (two-sample t-test p=0.02, Mann-Whitney U test p=0.03, n=24 and 21).

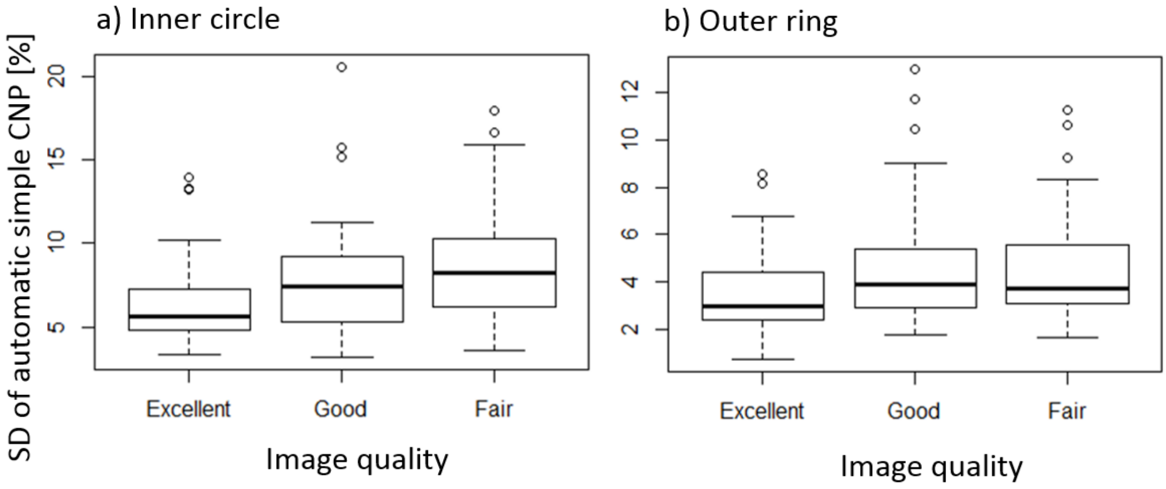

Figure 4 The variability of automatically calculated CNP positively correlates with the image quality (ANOVA, p<0.001, p=0.01, n=132). For each macula we calculated the CNP proportions for each of 24 sectors and then we calculated their standard deviation from the overall CNP.

	Image quality			
Outcome group	Excellent	Good	Fair	Total
Survived	37<br>32%	45<br>40%	32<br>28%	114<br>100%
Died	8<br>44%	8<br>44%	2<br>11%	18<br>100%
Total	45	53	34	132

Table 1 Image quality per outcome group. There is no association between outcome group and the image quality (p=0.27, Fisher exact test).

**Associations using images of excellent quality (n=45)**		**Automatic grading:**<br>Total percentage of CNP damaged pixels in inner circle and in outer ring	
		Continuous scale, %	p-value
Inner circle	Survived	Mean=18.0<br>SD=4.1	p=0.76 (Logistic regression)<br>p=0.72 (2-sample t-test)
	Died	Mean=19.1<br>SD=3.1%	
Outer ring	Survived	Mean=7.2%<br>SD=2.7%	p=0.34 (Logistic regression)<br>p=0.27 (2-sample t-test)
	Died	Mean=8.2%<br>SD=2.0%	

Table 2 Associations of simple CNP measures vs death using image of excellent quality.
